# Clusterin: a marker and mediator of chemoresistance in colorectal cancer

**DOI:** 10.1007/s10555-024-10173-y

**Published:** 2024-02-06

**Authors:** Sara Hlavca, Wing Hei Chan, Rebekah M. Engel, Helen E. Abud

**Affiliations:** 1 Department of Anatomy and Developmental Biology, Monash University, Clayton, VIC 3800 Australia; 2 Development and Stem Cells Program, Monash Biomedicine Discovery Institute, Clayton, VIC 3800 Australia; 3 Department of Surgery, Cabrini Monash University, Cabrini Hospital, Malvern, VIC 3144 Australia

**Keywords:** CLU, Colorectal cancer, Regenerative stem cells, Chemotherapy, Tumour relapse

## Abstract

Intra-tumoural heterogeneity and cancer cell plasticity in colorectal cancer (CRC) have been key challenges to effective treatment for patients. It has been suggested that a subpopulation of LGR5-expressing cancer stem cells (CSCs) is responsible for driving tumour relapse and therapy resistance in CRC. However, studies have revealed that the LGR5^+ve^ CSC population is highly sensitive to chemotherapy. It has been hypothesised that another subset of tumour cells can phenotypically revert to a stem-like state in response to chemotherapy treatment which replenishes the LGR5^+ve^ CSC population and maintains tumour growth. Recently, a unique stem cell population marked by enriched clusterin (CLU) expression and termed the revival stem cell (RevSC) was identified in the regenerating murine intestine. This CLU-expressing cell population is quiescent during homeostasis but has the ability to survive and regenerate other stem cells upon injury. More recently, the CLU^+ve^ signature has been implicated in several adverse outcomes in CRC, including chemotherapy resistance and poor patient survival; however, the mechanism behind this remains undetermined. In this review, we discuss recent insights on CLU in CRC and its roles in enhancing the plasticity of cells and further consider the implications of CLU as a prospective target for therapeutic intervention.

## Introduction

The development of malignancy along all sections of the colon or rectum is referred to as colorectal cancer (CRC). CRC presents a severe global health burden as the third most commonly diagnosed cancer and one of the top four leading causes of cancer-associated death [1–4]. Early stage CRC is often curable through surgical interventions; however, the survival rate for patients diagnosed with metastatic disease significantly drops to as low as 10% [5, 6]. Most of the deaths that occur from this disease are due to cancer recurrence at secondary sites after initial treatment [7] with tumour relapse and development of chemoresistance presenting a key challenge to the effective treatment of CRC.

Cellular plasticity, where cells can transition between different cell states, is a feature of solid tumours that contributes to tumour progression and relapse [8, 9]. Cellular phenotypes are dynamic and capable of responding to environmental pressures to control cell fate. This includes entering a quiescent stage upon challenge with anti-neoplastic agents that target dividing cells. The cancer stem cell (CSC) hypothesis is one model that describes cellular plasticity within tumours whereby a small subpopulation of tumour cells with regenerative potential drives tumour growth, therapy resistance, recurrence and metastasis [10]. This is evidenced by an association between the expression of stem cell markers and an increased risk of tumour recurrence [11]. Therefore, due to their ability to self-renew and differentiate, cells that possess stem cell properties within tumours represent a prospective target for therapeutic intervention. A key feature of stem cells in the normal intestinal epithelium is the ability to drive the continual production of cells and to restore the integrity of the epithelial lining following injury. Daily turnover of cells is driven by crypt base columnar (CBC) stem cells marked by leucine-rich repeat-containing G-protein coupled receptor 5 (LGR5) [12]. Upon injury, LGR5^+ve^ CBC stem cells are rapidly lost and replaced by LGR5^−ve^ cells [13, 14]. Key studies have revealed that the LGR5^+ve^ stem cells within CRC tumours are also sensitive to injury through radiation and chemotherapy [15–17]. It has been suggested that the LGR5^+ve^ CSC population is replenished after therapeutic treatment by progenitor cell types that revert into a stem-like state to drive tumour regeneration through mechanisms of cellular plasticity [17–20]. Furthermore, metastasis is primarily driven by LGR5^−ve^ tumour cells that have greater dissemination potential, inducing secondary tumour growth at distant sites through a process of dedifferentiation into LGR5^+ve^ tumour cells [20].

Recently, a unique revival stem cell (RevSC) population has been identified in regenerating murine epithelium by single-cell RNA sequencing [21]. The RevSC, characterised by high clusterin (CLU) expression, is a quiescent stem cell population during homeostasis [21]. However, upon tissue injury and subsequent upregulation of YAP signalling, CLU-expressing cells proliferate and restore the damaged epithelium, including LGR5^+ve^ CBC stem cells [21]. As LGR5^+ve^ CSCs share many of their characteristics with LGR5^+ve^ CBC cells and most likely originate from these CBC cells during cancer initiation [22, 23], it is possible that a regenerative stem cell type similar to the CLU^+ve^ RevSC is also present in the tumour tissue and may be either partly or entirely responsible for the repopulation of the CSC population following therapeutic treatment. High CLU expression has been associated with several adverse outcomes in CRC including poor patient prognosis, tumour metastasis and chemotherapy resistance [24–28]. Despite this, the underlying mechanisms are yet to be elucidated. In this review, we discuss recent advancements in our understanding of CLU in a cancer context and its possible roles in enhancing the plasticity of regenerative stem cells in CRC. We further consider the implications of CLU^+ve^ regenerative stem cells as a potential therapeutic target.

## Structure and function of the clusterin protein

**Fig. 1:**
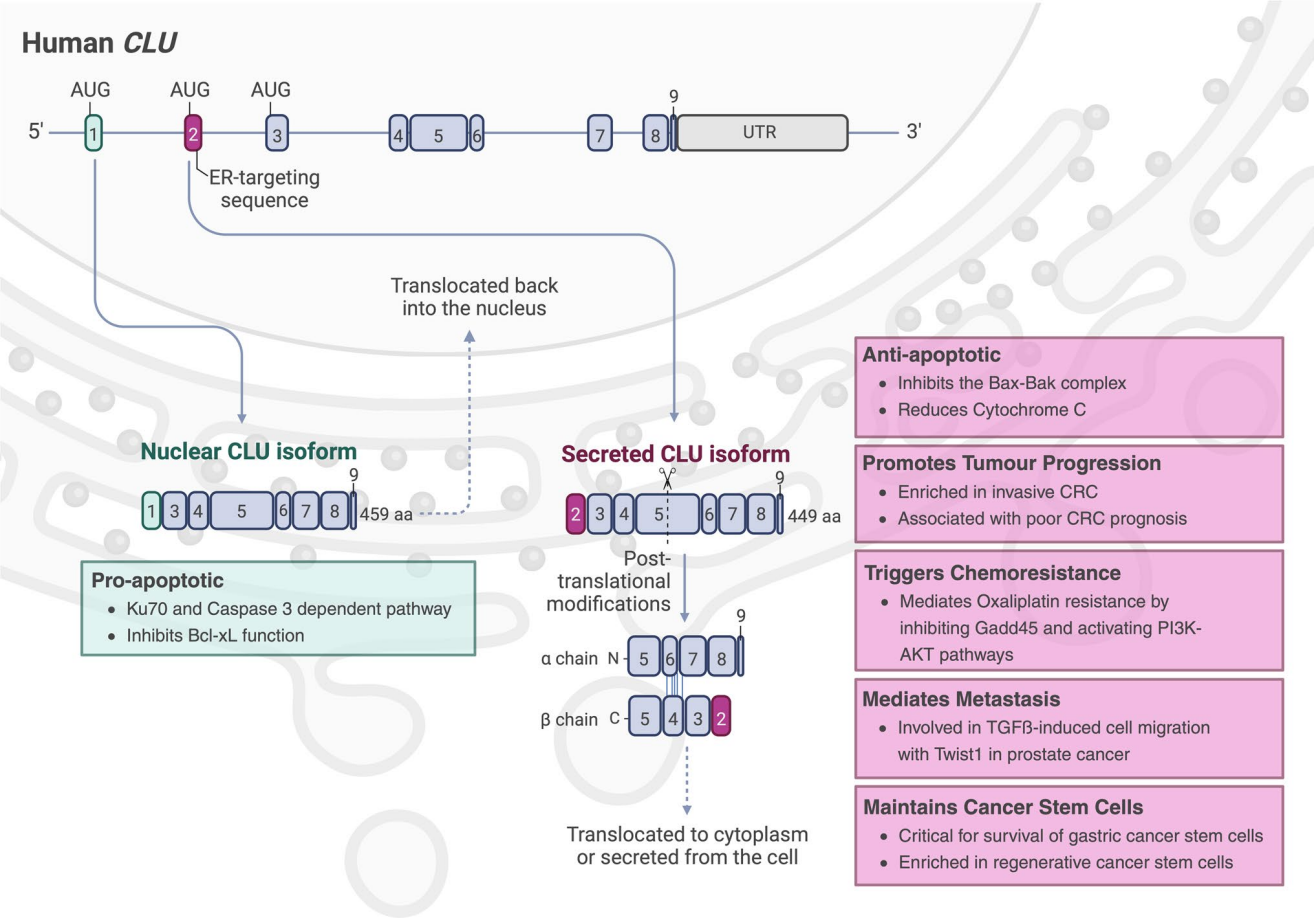
Structure and function of nuclear clusterin (nCLU) and secreted clusterin (sCLU) isoforms. The *CLU* gene is composed of nine exons. Two *CLU* isoforms are generated through alternative splicing where exon 2 is skipped for nCLU. For sCLU, translation begins at the AUG start codon in exon 2, whereas nCLU initiates in exon 3. These two isoforms have very distinct properties and are localised in different cellular compartments. nCLU is retained in the nucleus and promotes apoptosis either by affecting DNA repair through Ku70 binding [37, 46, 47], or by inhibiting the pro-apoptotic protein, Bcl-xL [48]. In contrast, sCLU translocates to the cytoplasm under stress or is secreted from the cell during homeostasis [38]. In the cytosol, sCLU inhibits oligomerisation of Bax, preventing the release of cytochrome c and apoptosis [42]. This results in tumour progression, chemotherapy resistance and promotion of metastasis. Furthermore, sCLU has been identified in cancer stem cells [49, 50], playing a critical role in aiding gastric cancer stem cell survival [50]; however its function and molecular mechanism within these cells remain to be elucidated

CLU, also referred to as apolipoprotein J (APOJ), sulphated glycoprotein 2 (SGP2), serum protein 40,40 (SP-40,40), X-ray-inducible transcript 8 (XIP8), complement lysis inhibitor (CLI) or testosterone-repressed prostate message 2 gene (TRPM-2) is a sulphated glycoprotein first discovered in 1979 in human salivary extract [29, 30]. In 1983, CLU was characterised and named due to its cell-aggregating ability [31]. CLU is expressed in various tissues and bodily fluids including the testes [32], brain [32], liver [32], kidney and thymus [33]. In humans, the *CLU* gene is located on position p21 of chromosome 8. The gene contains nine exons [34, 35] and encodes at least two protein isoforms: the conventional 70–80 kDa secreted CLU (sCLU) and the 49 kDa non-secreted nuclear CLU (nCLU) (Fig. 1). The sCLU is a heterodimer glycoprotein consisting of two 35–40 kDa subunits, the α-chain and β-chain, linked by five disulphide bonds [32, 36] (Fig. 1). nCLU, on the other hand, lacks the endoplasmic reticulum (ER)-targeting sequence due to exon 2 skipping by alternative splicing, therefore is translocated back to the nucleus upon translation without cleavage or glycosylation [37] (Fig. 1). Thus, these isoforms are present in distinct subcellular locations and perform different functions.

The sCLU isoform is typically secreted into the extracellular fluid; however, it is retained in the cytosol upon cellular stress [38] (Fig. 1). sCLU was first described as stress-induced chaperone, protecting cells by clearing misfolded proteins through hydrophobic interactions [39]. It has been implicated in various physiological processes including sperm maturation [40], complement inhibition [36], lipid transport [32] and inhibition of apoptosis [41]. One study reported a decrease in cell death, cytochrome c expression and caspase-9 activation in sCLU overexpressing HT1080 fibrosarcoma cells following treatment with a chemotherapeutic agent [42]. However, conditioned media produced from sCLU overexpressing HT1080 cells had no such effect on apoptosis. Therefore, it appears that extracellular sCLU does not possess the same anti-apoptotic function as cytosolic sCLU [42]. The CLU α-chain interacts with the Bcl-2-associated X (Bax) protein and prevents oligomerisation, and function of Bax [42]. Bax is required for the release of cytochrome c from the mitochondria to activate caspase-9, that in turn initiates apoptosis. The inhibition of this process by cytosolic CLU results in an anti-apoptotic function within the cell (Fig. 1).

Conversely, the nCLU isoform, which appears to be pro-apoptotic, lacks the hydrophobic ER-signal sequence typical of secreted proteins and instead translocates to the nuclear compartment within apoptotic cells [38, 43–45] (Fig. 1). nCLU was shown to bind to the 70 kDa protein of the Ku autoantigen (Ku70) through a Ku70-binding domain in the C-terminus [37, 46, 47]. Ku70 in turn forms a heterodimer with an 80 kDa protein (Ku80), which, in addition to DNA-dependent protein kinase, stimulates DNA repair [46, 47]. While the exact mechanisms of induction of apoptosis by nCLU through Ku70 are unclear, mutations in the Ku70 binding domain which prevent nCLU from binding to Ku70 result in decreased apoptosis in MCF-7 breast cancer cells [37]. Overexpression of nCLU in MCF-7 cells results in a reduction in cell growth due to an increase in cell death [46]. More recently, it was revealed that nCLU can bind directly via its C-terminal coiled-coil domain to B-cell lymphoma-extra large (Bcl-xL) [48]. Bcl-xL is itself an inhibitor of apoptosis, demonstrating that nCLU can indirectly promote apoptosis [48].

### Regulation of clusterin gene expression

**Fig. 2:**
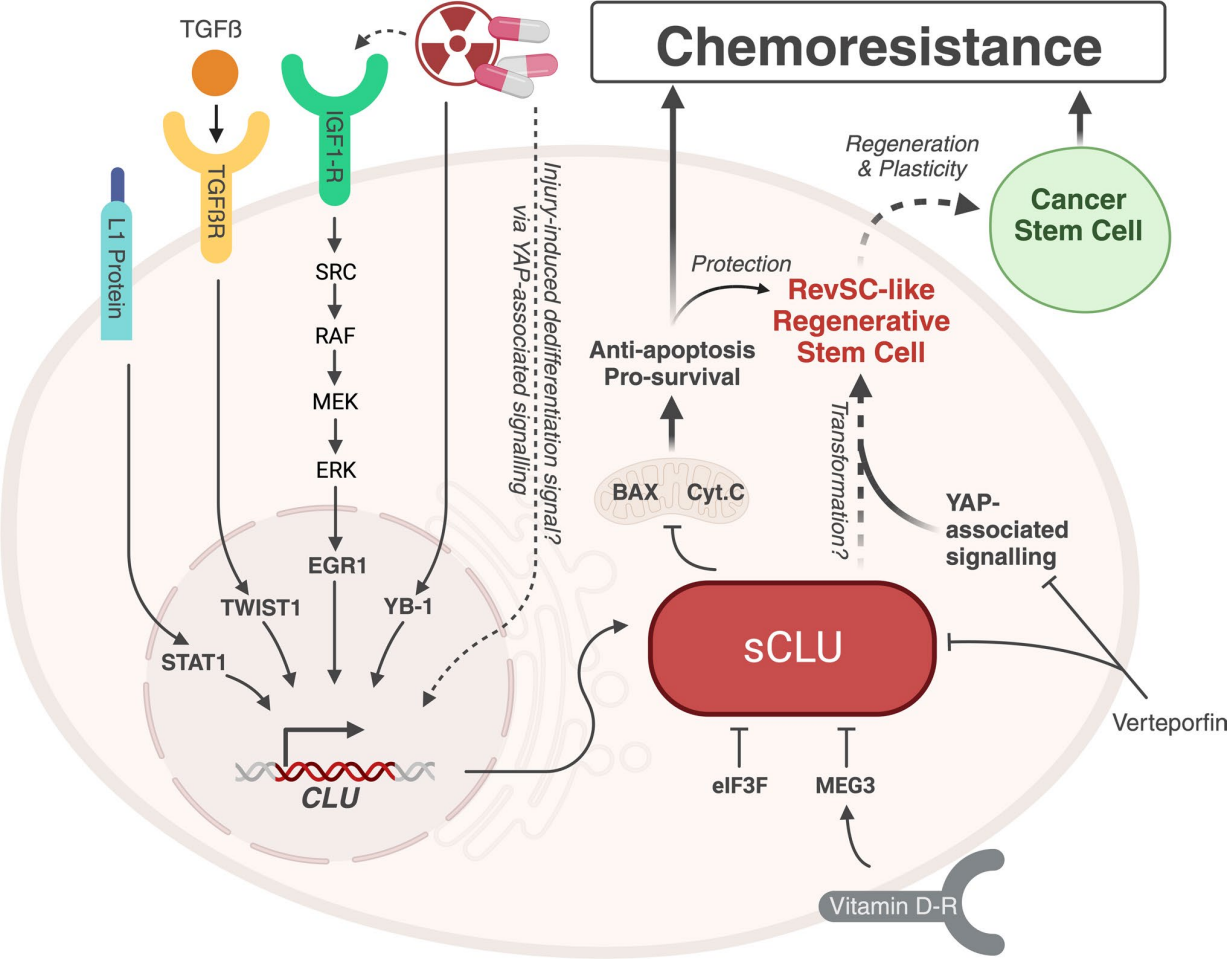
*CLU* expression can be activated by a variety of stimuli. L1CAM acts via STAT1 [28]; TGFβ activates TGFβR and TWIST1 [43, 51, 52, 60], IGF1-R and the downstream Src/Raf/Mek/Erk cascade acts via Egr-1 to activate expression of CLU [53]. Cellular injury caused by ionising radiation or chemotherapy has also been shown to induce *CLU* expression via the YB-1 In contrast, eIF3f [56] and MEG3 (via the vitamin D receptor) [57] can inhibit CLU action by interacting directly with sCLU. Pro-apoptotic Bax activity is inhibited by sCLU which promotes cell survival. CLU expression has more recently been associated with a unique RevSC population that is upregulated via YAP signalling following injury [21] which may define a cancer stem cell population that is resistant to chemotherapy in CRC

CLU expression is modulated by triggers including cellular stress and is downstream of several different signal transduction pathways (Fig. 2). Transforming growth factor β (TGFβ) signalling induces expression of CLU in CCL64 cells [43] via activation through AP-1-binding sites (5*-TGAGTCA) in the minimal promoter region of *CLU* [51, 52]. In MCF7 breast cancer cells, ionizing radiation-induced activation of the insulin-like growth factor (IGF)-1 receptor and the downstream Src/Raf/Mek/Erk cascade leads to *CLU* expression through early growth response 1 transactivation [53] (Fig. 2). The stress-activated transcription factor, Y-box binding protein-1 has also been reported to mediate CLU expression via direct binding to the promoter region following treatment with chemotherapeutic paclitaxel in prostate cancer cell lines [54] (Fig. 2). In CRC cell lines, the cell adhesion molecule L1 protein upregulates *CLU* levels through the binding of signal transducer and activator of transcription 1 (STAT-1) to the CLU promoter which is independent of NF-κB [28] (Fig. 2). In addition, histone modifications have also been linked to the regulation of expression of the CLU isoforms, with H3K4me3 and H3K9me3 enhancing nCLU expression by alternative splicing in CRC cell lines [55]. In contrast, the eukaryotic initiation factor 3 subunit f (eIF3f) has been shown to negatively affect CLU expression by directly interacting with the α-chain of sCLU and inhibiting its secretion [56] (Fig. 2). In addition to this, it has been found that vitamin D can act to inhibit *CLU* in CRC indirectly through a long non-coding RNA referred to as maternally expressed gene 3 *(MEG3)* [57] (Fig. 2). Finally, recent studies have revealed that intestinal cells enriched with YAP-transcriptional signatures express a high level of *CLU* [58, 59] and the activation of YAP1-dependent signalling induced ectopic CLU-expressing cells in the intestinal epithelium [21]. Conversely, treatment with verteporfin, an inhibitor of YAP-signalling, blocked CLU expression [50] (Fig. 2).

## Clusterin in colorectal cancer

High CLU expression has been associated with various cancers, including CRC [61]. Moreover, the abundance, intracellular localisation and histological distribution of CLU expression have been associated with the progression of CRC [38, 62–65] (Table 1). Conversely, a recent pan-cancer analysis by Fu and colleagues of *CLU* expression revealed decreased expression across most cancers compared to the matched normal tissue, including CRC [66]. However, this could be attributed to the opposing functions of the CLU isoforms which as previously mentioned, play very different roles in the cell. For example, elevated expression of sCLU has been reported in invasive colorectal adenocarcinomas, whereas expression in normal colonic tissues was detected only at very low levels [64]. Similar observations across different stages of colorectal tumorigenesis have also been reported, with 17% of the adenomas, 46% of the primary CRCs and 57% of the CRC metastatic lesions displaying overexpression of cytoplasmic CLU, compared to normal mucosa [62]. Whilst upregulation of CLU can occur in the early stages of premalignant adenomatous polyp formation, overexpression of CLU has been significantly correlated with advanced clinical stage [62]. High CLU expression also correlated with poor outcomes in stage II CRC within a cohort of 202 patients [63]. Additionally, sCLU was found to be abundant in the epithelium of tumour tissue, whereas in normal mucosa, sCLU is more abundant in the stroma [63]. Conversely, expression of nCLU was found to be decreased in colon cancer tissues compared to matched normal control tissues [67]. Whilst the nCLU isoform is pro-apoptotic and primarily expressed in normal colon epithelium, during tumour progression, expression of nCLU decreases with more sCLU translocated into the cytoplasm where it plays a protective role in preventing apoptosis [27, 68]. This shift in the expression of CLU isoforms is linked to increased tumour cell survival, aggressiveness and enhanced metastatic potential [69]. Therefore, these studies highlight an oncogenic role of sCLU during tumour progression and its potential as a diagnostic biomarker.

**Table 1:** Expression of clusterin (CLU) across normal, adenomatous and tumour tissue of colorectal origin

Studies	Diagnosis	TNM stage	CLU expression			Sample size
			Nucleus	Cytoplasm		
Chen et al. [64]	Normal colonic tissue	/	−	− (very weak)		2
	Hyperplastic polyps	/	−	+		3
	Tubular adenomas	/	+ + +	+ + +		1
	Villous adenomas	/	+ + +	+ + +		1
	Invasive adenocarcinomas	n/a	−	+ +		1
Pucci et al. [68]	Normal colonic tissue	/	+	−		30
	Adenoma	/	+	+		10
	Adenocarcinoma	I to II	−	+ + +		10
	Adenocarcinoma	III to IV	−	+ + + +		10
Xie et al. [62]	Normal colonic mucosa	/	n/a	+ (100%)		76
	Adenoma	/	n/a	+ + + (17%)		20
	Primary carcinoma	II	n/a	+ + + (33%)		42
	Primary carcinoma	III-IV	n/a	+ + + (60%)		43
	Metastases	IV	n/a	+ + + (57%)		35
Kevans et al. [63]				Epithelium	Stroma	
	Normal colonic mucosa	/	n/a	+	+ +	202
	Colorectal cancer	II	n/a	+ + +	+	202

TNM: Tumour, Nodes, Metastases; n/a: not available

### Clusterin expression as a diagnostic marker

Early-stage CRC is often treatable through surgical interventions and as such, early detection through the implementation of screening programs such as the faecal occult blood test has significantly reduced the burden of disease [70]. *CLU* expression has shown promise as a predictive biomarker for the identification of individuals at risk of developing CRC or in the early stages of disease [64, 71–74]. The progressive increase of sCLU expression in the setting of CRC correlates with a significant increase of CLU in the serum and stool of CRC patients [27, 73]. Moreover, Mazzarelli et al. [27] identified a significant positive correlation between CLU expression in stool and more advanced stage of disease [27]. In animal studies, it was also demonstrated that CLU secreted from a colon cancer cell line (Caco-2) injected into mice was detectable in blood samples, with the increasing level of CLU correlating with the increasing dimension of the tumours [72]. A positive correlation was identified between *CLU* expression and tumour severity with elevated expression of CLU also associated with a decrease in disease-free survival in CRC [24, 63, 65, 75] (Fig. 1). Again, Fu et al. [66] noted conflicting results from their pan-cancer analysis regarding *CLU* expression and overall survival, with high *CLU* expression conferring a survival advantage in some cancers, and a disadvantage to others (including CRC). Therefore, this suggests that monitoring expression levels of the sCLU isoform specifically may be a useful biomarker for the detection of disease as well as potential as a surveillance tool, in a similar approach to monitoring of circulating tumour DNA for the detection of disease relapse.

### Clusterin in chemoresistance and metastasis

In addition to its use as a diagnostic marker, high CLU expression is also associated with advanced tumours which are more prone to resist chemotherapy treatment and metastasis. Upregulated expression of CLU has been linked to increased chemoresistance in multiple cancer types including the breast, lung, prostate, bladder, liver, pancreatic, ovarian, cervical, melanoma and osteosarcoma [26, 38]. As the sCLU isoform has an anti-apoptotic function, the suppression of CLU expression may promote cell death when challenged by chemotherapy. Studies which modulate CLU expression have found that decreasing endogenous CLU expression through the means of drug [50, 53, 76], antisense oligonucleotide [77–81] or siRNA inhibition [82, 83] increases sensitivity to chemotherapeutics and reduces overall tumour burden (as reviewed in detail by Praharaj et al. [38]). In contrast, treatment with exogenous CLU results in increased resistance to chemotherapeutics [84], despite the fact that sCLU in conditioned media failing to demonstrate an anti-apoptotic effect in cell lines [42]. Combination therapy involving an antisense oligonucleotide, Custiren (OSX-011, an inhibitor of sCLU) [77, 79] with chemotherapeutics in various cancers, including prostate [85, 86], lung [87] and breast cancer [88] demonstrated improved patient survival in Phase II clinical trials. However, during Phase III synergy trials, OGX-011 combined with prednisone and cabazitaxel [89] or docetaxel [90] showed no significant improvement in overall survival in castration-resistant prostate cancer patient.

*CLU* expression has recently been examined in CRC patient-derived organoids (PDOs) that had been treated with the chemotherapeutic 5-FU [24]. *CLU* expression is not only significantly increased after 5 days of chemotherapy treatment *in vitro* but also correlated with an increase in PDO resistance to chemotherapy [24]. In hepatocellular carcinoma, sCLU was found to induce resistance to the chemotherapeutic oxaliplatin via activation of the phosphoinositide-3-kinase–protein kinase B (PI3K)/Akt pathway [91] (Fig. 1). Further analysis revealed that sCLU regulates PI3K/Akt pathway via downregulation of growth arrest and DNA-damage-inducible 45 alpha (Gadd45a) which itself decreases phosphorylation of Akt [92] (Fig. 1). Another study also explored the relationship between CLU expression and chemoresistance specifically in CRC by generating a SW480 CRC cell line that overexpresses intracellular sCLU. Interestingly, the sCLU overexpressing cells were more sensitive to combined chemotherapy treatment under normal and hypoxic conditions [93]. Therefore, further exploration of the role of CLU in therapy resistance in CRC is warranted, with consideration of factors including different cancer stages, mutation profiles and consensus molecular subtypes (CMSs) required.

In addition to chemoresistance, an increase in *CLU* expression has also been linked to metastasis. Flanagan et al. [25] generated a breast cancer cell line which overexpressed sCLU to explore the effect on treatment response. This study found that sCLU-overexpressing cells transplanted into host mice were more resistant to cytokine-induced apoptosis and were also more likely to metastasise to the lung compared to the parental cell line [25]. Moreover, upregulation of *CLU* expression combined with L1CAM mediated signalling, a marker of tumour cells with metastatic potential [94], within LS174T CRC tumour cells results in substantial metastasis formation within the liver and spleen after transplantation (Fig. 2) [28]. Subsequent suppression of *CLU* expression via shRNA significantly decreased the number and size of these metastases [28]. Similarly, silencing *CLU* expression via siRNA in SW480, SW620, and Caco2 CRC cell lines *in vitro* reduced their proliferative and migratory capabilities [95].

Furthermore, it was shown that *CLU* expression is necessary for TGFβ-induced cell migration as knockdown of both *CLU*, and its transcriptional regulator *Twist1*, significantly reduced the number of invasive prostate cancer cells [60] (Figs. 1, 2). This suggests that CLU may mediate epithelial-mesenchymal transition and thus metastasis formation through activation of the TGFβ signalling pathway. In contrast, overexpression of *MEG3* in CRC cell lines significantly inhibited cell proliferation and cell migration *in vitro* which corresponded to a reduction in tumour growth and metastasis formation in xenograft models [57].

## Clusterin-expressing regenerative stem cells

Enriched CLU expression marks the injury-induced stem cell type [21]. This damage-enriched CLU^+ve^ regenerative stem cell is highlighted for its tolerance to injury and cellular plasticity to regenerate damaged cells, and more recently has been identified in CRC (reviewed by Tape [96]). Upon irradiation or chemical-induced tissue injury, CLU^+ve^ cells can reconstitute all cell types within the intestinal crypt, including the LGR5^+ve^ CBC population [21]. The CLU^+ve^ RevSC is believed to be a unique stem cell population that is quiescent during homeostasis and proliferative upon tissue injury where these cells appear to revert to a fetal phenotype, with a transcriptional profile characterised by expression of *Anxa1*, *Ly6a (Sca1)* and *Clu* [21, 58, 59, 97]. In addition to this, the YAP gene signature is present exclusively in the CLU^+ve^ RevSC and not in the LGR5^+ve^ CBC population [21]. Knockout of YAP1 inhibits the expansion of CLU^+ve^ RevSCs after injury, while activation of YAP1 causes premature emergence of the CLU^+ve^ RevSCs under homeostasis confirming the dependence on YAP signalling [21]. As cytoplasmic CLU is known to play a pro-survival role in cells, this could be a possible explanation for the maintenance of these cells in response to injury. In support of this, depletion of CLU, or treatment with verteporfin, an inhibitor of CLU and YAP-signalling, in patient-derived gastric cancer tumorspheres causes apoptosis of gastric cancer stem cells and reduced tumour growth, highlighting the critical pro-survival and oncogenic role of CLU [50]. A recent functional single-cell study demonstrates that stromal TGFß1 and WNT3a produced from fibroblasts can enrich colonic RevSC via YAP signalling under low PI3K and MAPK signalling conditions [98]. This suggests that stromal signals via YAP could be potentially targeted, blocking the transition towards more drug-tolerant RevSCs in CRC. This highlights the intricate interplay of the oncogenic and stromal signalling in cell-fate plasticity during colonic oncogenesis.

A RevSC signature, that includes *CLU*, has been confirmed in human colorectal tumours and, similarly to what has been observed in the mouse intestine, it appears to be largely mutually exclusive with the LGR5^+ve^ stem cell signature [49]. This RevSC signature was also found to be upregulated in serrated tumours, which constitute 15–30% of all colorectal tumours, and harbour different mutational and epigenetic signatures to conventional colorectal tumours [99]. Conversely, the LGR5^+ve^ CSC signature was predominantly found in conventional colorectal tumours [49]. CRC can further be divided into four consensus molecular sub-types which categorise CRC tumours based on their molecular features including mutational signature, somatic copy number alterations, methylation status and proteomic profile [100]. The CLU^+ve^ RevSC signature has been identified primarily within CMS4 tumours, which are characterised by their mesenchymal phenotype due to strong stromal infiltration and have worse overall survival when compared to the other subtypes [49, 100, 101]. The LGR5^+ve^ CSC signature is instead overexpressed in CMS2 tumours which represent canonical activation of WNT signalling, once again highlighting the distinct expression of the two stem cell signatures [49, 100, 101].

Quiescent stem cell populations have been identified in PDOs derived from colorectal tumours [24, 102]. These cells are enriched for CLU and can re-enter the cell cycle and generate organoids when isolated through FACS [102]. This supports the notion that CRCs have considerable plasticity where a CLU^+ve^ cells can transition to a LGR5^+ve^ CSC phenotype or vice versa, depending on the environment and could form the basis of differential responses to treatments. This transitional signature is supported by further work on cell-state plasticity in the initiation of metastasis, whereby tumour cells transition from a LGR5^+ve^ CSC tumour characterised by canonical intestinal gene signatures into tumour cells expressing non-canonical gene pathways, which in turn were positively associated with metastasis and worse overall survival [103]. This transition between canonical and non-canonical states was further associated with a fetal progenitor signature which was highly expressed during the transition point and thus suggests a potential reversion to a fetal-like state prior to the acquisition of metastatic-promoting characteristics [103]. Therefore, the ability of tumour cells to adapt and shift phenotype not only affects a response to chemotherapy but may also increase the propensity for the tumour to metastasise. To address this, it is necessary to identify the key drivers of these stem cell populations and target both simultaneously to completely eradicate the tumour cells and prevent treatment resistance or disease relapse.

### The clusterin-expressing regenerative stem cell as a potential therapeutic target

Extensive evidence has linked an increase in CLU expression with an increase in tumour severity and treatment resistance. However, the mechanism underlying increased chemoresistance in CRC associated with high CLU expression remains unclear and may be due to the anti-apoptotic effect of CLU, the plasticity of CLU^+ve^ cells, or even the combination of both factors. It is evident that the pro-survival role of CLU indeed contributes to the maintenance of gastric CSCs in response to injury [50] and promotes CSC phenotype in hepatocellular carcinoma cells [104]. Whereas inhibition of CLU in breast CSCs has been shown to increase chemosensitivity through necrosis activation [105].

The RevSC signature, including *CLU*, has been implicated with an overall poor prognosis and it is primarily expressed within chemoresistant CRC tumour cells [49]. This signature is also observed immediately after ablation of the LGR5^+ve^ CSCs in mouse models of CRC, followed by repopulation of the LGR5^+ve^ CSC 5 days later [49]. An increase in CLU expression, as well as other RevSC markers including *Anxa1* and *Basp1*, is also evident following chemotherapy treatment in genetically engineered mouse organoid models of CRC [106], and this correlates with what is observed in CRC PDOs [24]. Expression of these RevSC markers is subsequently reduced once chemotherapy is withdrawn and the organoids have recovered [106]. This demonstrates that the model of CLU^+ve^ cells repopulating LGR5^+ve^ CSCs after loss or damage can also be observed *in vitro.* Therefore, the CLU^+ve^ regenerative stem cell is emerging as a potential therapeutic target for the prevention of chemo- and radiotherapy resistance and tumour relapse. However, the mechanisms and processes underlying this are yet to be discovered, and the role of CLU in regulating the function of regenerative stem cells is still undetermined.

Interestingly, a correlation between the RevSC and TGFβ signalling (a key regulator of *CLU* expression) has been observed by several groups, with upregulated TGFβ pathway activation in RevSC-enriched tumours. A work by Fodde et al. [107] specifically notes a correlation between increased *Clu* expression and TGFβ signalling in mouse colorectal tumours which develop through an inflammatory pathway [107]. Additionally, treatment with TGFβ pushes mouse WT and tumour organoids towards a RevSC phenotype [49], while organoids co-cultured with mesenchymal cells pre-treated with TGFβ1 exhibit higher expression of RevSC genes, including *Clu*, compared to organoids co-cultured with vehicle pre-treated mesenchyme [108]. Another study by Sharif et al. [109] identifies a mesenchymal-derived signal (Asporin) which is induced transiently after chemotherapy treatment and drives an upregulation of *Clu* expression via the Tgfb receptor [109]. Subsequent inhibition of the TGFβ receptor via A8301 prevents this increase in *Clu* expression and the organoids lose their regenerative phenotype (represented by a cystic shape and decreased budding) [109]. As TGFβ signalling is known to regulate *CLU* expression, this provides a potential avenue for CLU to regulate the function of the RevSC, particularly given its known role as an inhibitor of apoptosis.

## Conclusion and future directions

The tumour stem cell population is no longer considered a static population but has instead been shown to adapt and shift phenotype in response to environmental stresses. This is particularly relevant during chemotherapy treatment, where the sensitive LGR5^+ve^ CSC population is replenished by the residual resistant tumour cells, to reinitiate tumour formation. Hence, many therapeutics that fail to completely eradicate the whole tumour will ultimately result in relapse. The recent identification of a new regenerative stem cell population in CRC has provided a potential druggable target that may provide an avenue to improve treatment outcomes. CLU has been identified as a key marker of this regenerative stem cell, with clear associations with resistance to treatment and metastasis. This may be due to the anti-apoptotic function of CLU, or to the role of CLU in defining plasticity of cells within CRC. In particular, it remains to be determined if the *CLU* is important in the regenerative process (particularly as key pathways regulating the RevSC such as YAP and TGFβ have also previously been associated with regulation of *CLU* expression), or if it is simply a biomarker for this stem cell population. Regardless, the identification of the RevSC as the regenerative source of the LGR5^+ve^ CSC population following chemotherapy treatment, and the role *CLU* has in this process, could elucidate a druggable target that may prove efficacious for the treatment of CRC.

## References

[1] Favoriti P, Carbone G, Greco M, Pirozzi F, Pirozzi REM, Corcione F (2016). Worldwide burden of colorectal cancer: A review. Updates in surgery.

[2] Araghi M, Soerjomataram I, Jenkins M, Brierley J, Morris E, Bray F, Arnold M (2019). Global trends in colorectal cancer mortality: Projections to the year 2035. International Journal of Cancer.

[3] Center, M. M., Jemal, A., Smith, R. A., & Ward, E. (2009). Worldwide variations in colorectal cancer. *CA: A Cancer Journal for Clinicians, 59*(6), 366–378. 10.3322/caac.20038 10.3322/caac.20038 19897840

[4] WHO. (2023, 11 July). *Colorectal cancer*. World Heath Organisation. Retrieved September 14, 2023 from https://www.who.int/news-room/fact-sheets/detail/colorectal-cancer#:~:text=The%20risk%20of%20colorectal%20cancer,early%20stages%20of%20the%20disease

[5] Munro MJ, Wickremesekera SK, Peng L, Tan ST, Itinteang T (2018). Cancer stem cells in colorectal cancer: A review. Journal of Clinical Pathology.

[6] Riihimäki M, Hemminki A, Sundquist J, Hemminki K (2016). Patterns of metastasis in colon and rectal cancer. Scientific Reports.

[7] Lintoiu-Ursut, B., Tulin, A., & Constantinoiu, S. (2015). Recurrence after hepatic resection in colorectal cancer liver metastasis - Review article.* Journal of Medicine & Life, 8*(Special Issue 1), 12–14. PMC4564049 26361505

[8] Massagué J, Ganesh K (2021). Metastasis-initiating cells and ecosystems. Cancer Discovery.

[9] Gupta PB, Pastushenko I, Skibinski A, Blanpain C, Kuperwasser C (2019). Phenotypic plasticity: Driver of cancer initiation, progression, and therapy resistance. Cell Stem Cell.

[10] Batlle E, Clevers H (2017). Cancer stem cells revisited. Nature Publishing Group.

[11] Merlos-Suarez A, Barriga FM, Jung P, Iglesias M, Cespedes MV, Rossell D, Sevillano M, Hernando-Momblona X, Silva-Diz VD, Munoz P, Clevers H, Sancho E, Mangues R, Batlle E (2011). The intestinal stem cell signature identifies colorectal cancer stem cells and predicts disease relapse. Stem Cell.

[12] Barker N, van Es JH, Kuipers J, Kujala P, van den Born M, Cozijnsen M, Haegebarth A, Korving J, Begthel H, Peters PJ, Clevers H (2007). Identification of stem cells in small intestine and colon by marker gene Lgr5. Nature.

[13] de Sousa e Melo F, de Sauvage FJ (2019). Cellular plasticity in intestinal homeostasis and disease. Cell stem cell.

[14] Davidson LA, Goldsby JS, Callaway ES, Shah MS, Barker N, Chapkin RS (2012). Alteration of colonic stem cell gene signatures during the regenerative response to injury. Biochimica et Biophysica Acta (BBA) - Molecular Basis of Disease.

[15] Planutis AK, Holcombe RF, Planoutene MV, Planoutis KS (2015). SW480 colorectal cancer cells that naturally express Lgr5 are more sensitive to the most common chemotherapeutic agents than Lgr5-negative SW480 cells. Anti-Cancer Drugs.

[16] Metcalfe C, Kljavin NM, Ybarra R, Sauvage FJD (2013). Lgr5+ stem cells are indispensable for radiation-induced intestinal regeneration. Cell stem cell.

[17] Asfaha S, Hayakawa Y, Muley A, Stokes S, Graham TA, Ericksen RE, Westphalen CB, Burstin JV, Mastracci TL, Worthley DL, Guha C, Quante M, Rustgi AK, Wang TC (2015). Krt19(+)/Lgr5(-) cells are radioresistant cancer-initiating stem cells in the colon and intestine. Cell stem cell.

[18] Shimokawa M, Ohta Y, Nishikori S, Matano M, Takano A, Fujii M, Date S, Sugimoto S, Kanai T, Sato T (2017). Visualization and targeting of LGR5+ human colon cancer stem cells. Nature.

[19] Tetteh PW, Basak O, Farin HF, Wiebrands K, Kretzschmar K, Begthel H, van den Born M, Korving J, de Sauvage F, van Es JH, van Oudenaarden A, Clevers H (2016). Replacement of lost Lgr5-positive stem cells through plasticity of their enterocyte-lineage daughters. Cell Stem Cell.

[20] Fumagalli A, Oost KC, Kester L, Morgner J, Bornes L, Bruens L, Spaargaren L, Azkanaz M, Schelfhorst T, Beerling E, Heinz MC, Postrach D, Seinstra D, Sieuwerts AM, Martens JWM, van der Elst S, van Baalen M, Bhowmick D, Vrisekoop N, Ellenbroek SIJ, Suijkerbuijk SJE, Snippert HJ, van Rheenen J (2020). Plasticity of Lgr5-negative cancer cells drives metastasis in colorectal cancer. Cell Stem Cell.

[21] Ayyaz A, Kumar S, Sangiorgi B, Ghoshal B, Gosio J, Ouladan S, Fink M, Barutcu S, Trcka D, Shen J, Chan K, Wrana JL, Gregorieff A (2019). Single-cell transcriptomes of the regenerating intestine reveal a revival stem cell. Nature Publishing Group.

[22] Huels DJ, Sansom OJ (2015). Stem vs non-stem cell origin of colorectal cancer. British Journal of Cancer.

[23] Hirata A, Hatano Y, Niwa M, Hara A, Tomita H (2019). Heterogeneity in colorectal cancer stem cells. Cancer Prevention Research.

[24] Engel RM, Chan WH, Nickless D, Hlavca S, Richards E, Kerr G, Oliva K, McMurrick PJ, Jardé T, Abud HE (2020). Patient-derived colorectal cancer organoids upregulate revival stem cell marker genes following chemotherapeutic treatment. Journal of clinical medicine.

[25] Flanagan L, Whyte L, Chatterjee N, Tenniswood M (2010). Effects of clusterin over-expression on metastatic progression and therapy in breast cancer. BMC Cancer.

[26] García-Aranda M, Téllez T, Muñoz M, Redondo M (2017). Clusterin inhibition mediates sensitivity to chemotherapy and radiotherapy in human cancer. Anti-Cancer Drugs.

[27] Mazzarelli, P., Pucci, S., & Spagnoli, L. G. (2009). CLU and colon cancer. The dual face of CLU. In *Clusterun, Part A* (Vol. 104, pp. 45–61). Elsevier Science and Technology. 10.1016/S0065-230X(09)05003-9 10.1016/S0065-230X(09)05003-9 19879422

[28] Shapiro B, Tocci P, Haase G, Gavert N, Ben-Ze’ev A (2015). Clusterin, a gene enriched in intestinal stem cells, is required for L1-mediated colon cancer metastasis. Oncotarget.

[29] Hogg SD, Embery G (1979). The isolation and partial characterization of a sulphated glycoprotein from human whole saliva which aggregates strains of Streptococcus sanguis but not Streptococcus mutans. Archives of Oral Biology.

[30] Rohne P, Prochnow H, Koch-Brandt C (2016). The CLU-files: Disentanglement of a mystery. Biomolecular Concepts.

[31] Fritz IB, Burdzy K, Sétchell B, Blaschuk O (1983). Ram rete testis fluid contains a protein (clusterin) which influences cell-cell interactions in vitro. Biology of Reproduction.

[32] de Silva HV, Harmony JA, Stuart WD, Gil CM, Robbins J (1990). Apolipoprotein J: Structure and tissue distribution. Biochemistry.

[33] French LE, Sappino AP, Tschopp J, Schifferli JA (1992). Distinct sites of production and deposition of the putative cell death marker clusterin in the human thymus. Journal of Clinical Investigation.

[34] Fink TM, Zimmer M, Tschopp J, Etienne J, Jenne DE, Lichter P (1993). Human clusterin (CLI) maps to 8p21 in proximity to the lipoprotein lipase (LPL) gene. Genomics.

[35] Andersen CL, Schepeler T, Thorsen K, Birkenkamp-Demtroder K, Mansilla F, Aaltonen LA, Laurberg S, Orntoft TF (2007). Clusterin expression in normal mucosa and colorectal cancer. Molecular & Cellular Proteomics.

[36] Jenne DE, Tschopp J (1989). Molecular structure and functional characterization of a human complement cytolysis inhibitor found in blood and seminal plasma: Identity to sulfated glycoprotein 2, a constituent of rat testis fluid. Proceedings of the National Academy of Sciences of the United States of America.

[37] Leskov KS, Klokov DY, Li J, Kinsella TJ, Boothman DA (2003). Synthesis and functional analyses of nuclear clusterin, a cell death protein. Journal of Biological Chemistry.

[38] Praharaj PP, Patra S, Panigrahi DP, Patra SK, Bhutia SK (2021). Clusterin as modulator of carcinogenesis: A potential avenue for targeted cancer therapy. Biochimica et Biophysica Acta (BBA) - Reviews on Cancer.

[39] Humphreys DT, Carver JA, Easterbrook-Smith SB, Wilson MR (1999). Clusterin has chaperone-like activity similar to that of small heat shock proteins. Journal of Biological Chemistry.

[40] Sylvester SR, Skinner MK, Griswold MD (1984). A sulfated glycoprotein synthesized by Sertoli cells and by epididymal cells is a component of the sperm membrane. Biology of Reproduction.

[41] Sensibar JA, Sutkowski DM, Raffo A, Buttyan R, Griswold MD, Sylvester SR, Kozlowski JM, Lee C (1995). Prevention of cell death induced by tumor necrosis factor alpha in LNCaP cells by overexpression of sulfated glycoprotein-2 (clusterin). Cancer Research.

[42] Zhang H, Kim JK, Edwards CA, Xu Z, Taichman R, Wang CY (2005). Clusterin inhibits apoptosis by interacting with activated Bax. Nature Cell Biology.

[43] Reddy KB, Jin G, Karode MC, Harmony JA, Howe PH (1996). Transforming growth factor beta (TGF beta)-induced nuclear localization of apolipoprotein J/clusterin in epithelial cells. Biochemistry.

[44] O'Sullivan J, Whyte L, Drake J, Tenniswood M (2003). Alterations in the post-translational modification and intracellular trafficking of clusterin in MCF-7 cells during apoptosis. Cell Death & Differentiation.

[45] Caccamo AE, Scaltriti M, Caporali A, D'Arca D, Scorcioni F, Candiano G, Mangiola M, Bettuzzi S (2003). Nuclear translocation of a clusterin isoform is associated with induction of anoikis in SV40-immortalized human prostate epithelial cells. Annals of the New York Academy of Sciences.

[46] Yang CR, Leskov K, Hosley-Eberlein K, Criswell T, Pink JJ, Kinsella TJ, Boothman DA (2000). Nuclear clusterin/XIP8, an x-ray-induced Ku70-binding protein that signals cell death. Proceedings of the National Academy of Sciences of the United States of America.

[47] Leskov K, Criswell T, Antonio S, Li J, Yang C-R, Kinsella T, Boothman D (2001). When X-ray-inducible proteins meet DNA double strand break repair. Seminars in Radiation Oncology.

[48] Kim N, Yoo JC, Han JY, Hwang EM, Kim YS, Jeong EY, Sun C-H, Yi G-S, Roh GS, Kim HJ, Kang SS, Cho GJ, Park J-Y, Choi WS (2012). Human nuclear clusterin mediates apoptosis by interacting with Bcl-XL through C-terminal coiled coil domain. Journal of Cellular Physiology.

[49] Vasquez EG, Nasreddin N, Valbuena GN, Mulholland EJ, Belnoue-Davis HL, Eggington HR, Schenck RO, Wouters VM, Wirapati P, Gilroy K, Lannagan TRM, Flanagan DJ, Najumudeen AK, Omwenga S, McCorry AMB, Easton A, Koelzer VH, East JE, Morton D, Trusolino L, Maughan T, Campbell AD, Loughrey MB, Dunne PD, Tsantoulis P, Huels DJ, Tejpar S, Sansom OJ, Leedham SJ (2022). Dynamic and adaptive cancer stem cell population admixture in colorectal neoplasia. Cell Stem Cell.

[50] Xiong J, Wang S, Chen T, Shu X, Mo X, Chang G, Chen J-J, Li C, Luo H, Lee J-D (2019). Verteporfin blocks Clusterin which is required for survival of gastric cancer stem cell by modulating HSP90 function. International Journal of Biological Sciences.

[51] Jin G, Howe PH (1997). Regulation of clusterin gene expression by transforming growth factor beta. Journal of Biological Chemistry.

[52] Wong P, Taillefer D, Lakins J, Pineault J, Chader G, Tenniswood M (1994). Molecular characterization of human TRPM-2/clusterin, a gene associated with sperm maturation, apoptosis and neurodegeneration. European Journal of Biochemistry.

[53] Criswell T, Beman M, Araki S, Leskov K, Cataldo E, Mayo LD, Boothman DA (2005). Delayed activation of insulin-like growth factor-1 receptor/Src/MAPK/Egr-1 signaling regulates clusterin expression, a pro-survival factor. Journal of Biological Chemistry.

[54] Shiota M, Zoubeidi A, Kumano M, Beraldi E, Naito S, Nelson CC, Sorensen PH, Gleave ME (2011). Clusterin is a critical downstream mediator of stress-induced YB-1 transactivation in prostate cancer. Molecular Cancer Research.

[55] Deb M, Sengupta D, Rath SK, Kar S, Parbin S, Shilpi A, Pradhan N, Bhutia SK, Roy S, Patra SK (2015). Clusterin gene is predominantly regulated by histone modifications in human colon cancer and ectopic expression of the nuclear isoform induces cell death. Biochimica et Biophysica Acta.

[56] Lee JY, Kim HJ, Rho SB, Lee SH (2016). eIF3f reduces tumor growth by directly interrupting clusterin with anti-apoptotic property in cancer cells. Oncotarget.

[57] Zhu Y, Chen P, Gao Y, Ta N, Zhang Y, Cai J, Zhao Y, Liu S, Zheng J (2018). MEG3 activated by vitamin D inhibits colorectal cancer cells proliferation and migration via regulating clusterin. eBioMedicine.

[58] Yui S, Azzolin L, Maimets M, Pedersen MT, Fordham RP, Hansen SL, Larsen HL, Guiu J, Alves MRP, Rundsten CF, Johansen JV, Li Y, Madsen CD, Nakamura T, Watanabe M, Nielsen OH, Schweiger PJ, Piccolo S, Jensen KB (2018). YAP/TAZ-dependent reprogramming of colonic epithelium links ECM remodeling to tissue regeneration. Cell Stem Cell.

[59] Pikkupeura LM, Bressan RB, Guiu J, Chen Y, Maimets M, Mayer D, Schweiger PJ, Hansen SL, Maciag GJ, Larsen HL, Lõhmussaar K, Pedersen MT, Teves JMY, Bornholdt J, Benes V, Sandelin A, Jensen KB (2023). Transcriptional and epigenomic profiling identifies YAP signaling as a key regulator of intestinal epithelium maturation. Science Advances.

[60] Shiota M, Zardan A, Takeuchi A, Kumano M, Beraldi E, Naito S, Zoubeidi A, Gleave ME (2012). Clusterin mediates TGF-β–induced epithelial–mesenchymal transition and metastasis via Twist1 in prostate cancer cells. Cancer Research.

[61] Shannan B, Seifert M, Leskov K, Willis J, Boothman D, Tilgen W, Reichrath J (2006). Challenge and promise: Roles for clusterin in pathogenesis, progression and therapy of cancer. Cell Death & Differentiation.

[62] Xie D, Sham JS, Zeng WF, Che LH, Zhang M, Wu HX, Lin HL, Wen JM, Lau SH, Hu L, Guan XY (2005). Oncogenic role of clusterin overexpression in multistage colorectal tumorigenesis and progression. World Journal of Gastroenterology.

[63] Kevans D, Foley J, Tenniswood M, Sheahan K, Hyland J, O'Donoghue D, Mulcahy H, O'Sullivan J (2009). High clusterin expression correlates with a poor outcome in stage II colorectal cancers. Cancer Epidemiology, Biomarkers & Prevention.

[64] Chen X, Halberg RB, Ehrhardt WM, Torrealba J, Dove WF (2003). Clusterin as a biomarker in murine and human intestinal neoplasia. Proceedings of the National Academy of Sciences of the United States of America.

[65] Artemaki PI, Sklirou AD, Kontos CK, Liosi A-A, Gianniou DD, Papadopoulos IN, Trougakos IP, Scorilas A (2020). High clusterin (CLU) mRNA expression levels in tumors of colorectal cancer patients predict a poor prognostic outcome. Clinical Biochemistry.

[66] Fu Y, Du Q, Cui T, Lu Y, Niu G (2022). A pan-cancer analysis reveals role of clusterin (CLU) in carcinogenesis and prognosis of human tumors. Frontiers in Genetics.

[67] Chen T, Turner J, McCarthy S, Scaltriti M, Bettuzzi S, Yeatman TJ (2004). Clusterin-mediated apoptosis is regulated by adenomatous polyposis coli and is p21 dependent but p53 independent. Cancer Research.

[68] Pucci S, Bonanno E, Pichiorri F, Angeloni C, Spagnoli LG (2004). Modulation of different clusterin isoforms in human colon tumorigenesis. Oncogene.

[69] Rodríguez-Piñeiro AM, de la Cadena MP, López-Saco Á, Rodríguez-Berrocal FJ (2006). Differential expression of serum clusterin isoforms in colorectal cancer. Molecular & Cellular Proteomics.

[70] Mendis S, Hong W, Ananda S, Faragher I, Jones I, Croxford M, Steel M, Jalali A, Gard G, To YH, Lee M, Kosmider S, Wong R, Tie J, Gibbs P (2022). Biology and clinical implications of fecal occult blood test screen-detected colorectal cancer. JNCI Cancer Spectrum.

[71] Bertuzzi M, Marelli C, Bagnati R, Colombi A, Fanelli R, Saieva C, Ceroti M, Bendinelli B, Caini S, Airoldi L, Palli D (2015). Plasma clusterin as a candidate pre-diagnosis marker of colorectal cancer risk in the Florence cohort of the European Prospective Investigation into Cancer and Nutrition: A pilot study. BMC Cancer.

[72] Pucci S, Bonanno E, Sesti F, Mazzarelli P, Mauriello A, Ricci F, Zoccai GB, Rulli F, Galata G, Spagnoli LG (2009). Clusterin in stool: A new biomarker for colon cancer screening?. The American Journal of Gastroenterology.

[73] Rodriguez-Pineiro AM, Garcia-Lorenzo A, Blanco-Prieto S, Alvarez-Chaver P, Rodriguez-Berrocal FJ, Cadena MP, Martinez-Zorzano VS (2012). Secreted clusterin in colon tumor cell models and its potential as diagnostic marker for colorectal cancer. Cancer Investigation.

[74] Strohkamp S, Gemoll T, Humborg S, Hartwig S, Lehr S, Freitag-Wolf S, Becker S, Franzen B, Pries R, Wollenberg B, Roblick UJ, Bruch HP, Keck T, Auer G, Habermann JK (2018). Protein levels of clusterin and glutathione synthetase in platelets allow for early detection of colorectal cancer. Cellular & Molecular Life Sciences.

[75] Redondo M, Rodrigo I, Alcaide J, Tellez T, Roldan MJ, Funez R, Diaz-Martin A, Rueda A, Jimenez E (2010). Clusterin expression is associated with decreased disease-free survival of patients with colorectal carcinomas. Histopathology.

[76] Mustafi S, Sant DW, Liu Z-J, Wang G (2017). Ascorbate induces apoptosis in melanoma cells by suppressing Clusterin expression. Scientific Reports.

[77] Chi KN, Zoubeidi A, Gleave ME (2008). Custirsen (OGX-011): A second-generation antisense inhibitor of clusterin for the treatment of cancer. Expert Opinion on Investigational Drugs.

[78] Zellweger T, Miyake H, Cooper S, Chi K, Conklin BS, Monia BP, Gleave ME (2001). Antitumor activity of antisense clusterin oligonucleotides is improved in vitro and in vivo by incorporation of 2'-O-(2-methoxy)ethyl chemistry. Journal of Pharmacology and Experimental Therapeutics.

[79] Zielinski R, Chi KN (2012). Custirsen (OGX-011): A second-generation antisense inhibitor of clusterin in development for the treatment of prostate cancer. Future Oncology.

[80] Chen Q, Wang Z, Zhang K, Liu X, Cao W, Zhang L, Zhang S, Yan B, Wang Y, Xia C (2011). Clusterin confers gemcitabine resistance in pancreatic cancer. World Journal of Surgical Oncology.

[81] Tang Y, Liu F, Zheng C, Sun S, Jiang Y (2012). Knockdown of clusterin sensitizes pancreatic cancer cells to gemcitabine chemotherapy by ERK1/2 inactivation. Journal of Experimental & Clinical Cancer Research.

[82] Yamamoto Y, Lin PJ, Beraldi E, Zhang F, Kawai Y, Leong J, Katsumi H, Fazli L, Fraser R, Cullis PR, Gleave M (2015). siRNA lipid nanoparticle potently silences clusterin and delays progression when combined with androgen receptor cotargeting in enzalutamide-resistant prostate cancer. Clinical Cancer Research.

[83] Park DC, Yeo SG, Shin EY, Mok SC, Kim DH (2006). Clusterin confers paclitaxel resistance in cervical cancer. Gynecologic Oncology.

[84] Park DC, Yeo SG, Wilson MR, Yerbury JJ, Kwong J, Welch WR, Choi YK, Birrer MJ, Mok SC, Wong KK (2008). Clusterin interacts with Paclitaxel and confer Paclitaxel resistance in ovarian cancer. Neoplasia.

[85] Chi KN, Eisenhauer E, Fazli L, Jones EC, Goldenberg SL, Powers J, Tu D, Gleave ME (2005). A phase I pharmacokinetic and pharmacodynamic study of OGX-011, a 2'-methoxyethyl antisense oligonucleotide to clusterin, in patients with localized prostate cancer. Journal of the National Cancer Institute.

[86] Saad F, Hotte S, North S, Eigl B, Chi K, Czaykowski P, Wood L, Pollak M, Berry S, Lattouf JB, Mukherjee SD, Gleave M, Winquist E (2011). Randomized phase II trial of Custirsen (OGX-011) in combination with docetaxel or mitoxantrone as second-line therapy in patients with metastatic castrate-resistant prostate cancer progressing after first-line docetaxel: CUOG trial P-06c. Clinical Cancer Research.

[87] Laskin JJ, Nicholas G, Lee C, Gitlitz B, Vincent M, Cormier Y, Stephenson J, Ung Y, Sanborn R, Pressnail B, Nugent F, Nemunaitis J, Gleave ME, Murray N, Hao D (2012). Phase I/II trial of custirsen (OGX-011), an inhibitor of clusterin, in combination with a gemcitabine and platinum regimen in patients with previously untreated advanced non-small cell lung cancer. Journal of Thoracic Oncology.

[88] Chia S, Dent S, Ellard S, Ellis PM, Vandenberg T, Gelmon K, Powers J, Walsh W, Seymour L, Eisenhauer EA (2009). Phase II trial of OGX-011 in combination with docetaxel in metastatic breast cancer. Clinical Cancer Research.

[89] Beer TM, Hotte SJ, Saad F, Alekseev B, Matveev V, Flechon A, Gravis G, Joly F, Chi KN, Malik Z, Blumenstein B, Stewart PS, Jacobs CA, Fizazi K (2017). Custirsen (OGX-011) combined with cabazitaxel and prednisone versus cabazitaxel and prednisone alone in patients with metastatic castration-resistant prostate cancer previously treated with docetaxel (AFFINITY): A randomised, open-label, international, phase 3 trial. Lancet Oncology.

[90] Chi KN, Higano CS, Blumenstein B, Ferrero JM, Reeves J, Feyerabend S, Gravis G, Merseburger AS, Stenzl A, Bergman AM, Mukherjee SD, Zalewski P, Saad F, Jacobs C, Gleave M, de Bono JS (2017). Custirsen in combination with docetaxel and prednisone for patients with metastatic castration-resistant prostate cancer (SYNERGY trial): A phase 3, multicentre, open-label, randomised trial. Lancet Oncology.

[91] Xiu P, Dong X, Dong X, Xu Z, Zhu H, Liu F, Wei Z, Zhai B, Kanwar JR, Jiang H, Li J, Sun X (2013). Secretory clusterin contributes to oxaliplatin resistance by activating Akt pathway in hepatocellular carcinoma. Cancer Science.

[92] Wang X, Zou F, Zhong J, Yue L, Wang F, Wei H, Yang G, Jin T, Dong X, Li J, Xiu P (2018). Secretory clusterin mediates Oxaliplatin resistance via the Gadd45a/PI3K/Akt signaling pathway in hepatocellular carcinoma. Journal of Cancer.

[93] Kevans D, Gorman S, Tosetto M, Sheahan K, O'Donoghue D, Mulcahy H, O'Sullivan J (2012). Clusterin and chemotherapy sensitivity under normoxic and graded hypoxic conditions in colorectal cancer. Journal of Gastrointestinal Cancer.

[94] Ganesh K, Basnet H, Kaygusuz Y, Laughney AM, He L, Sharma R, O'Rourke KP, Reuter VP, Huang YH, Turkekul M, Er EE, Masilionis I, Manova-Todorova K, Weiser MR, Saltz LB, Garcia-Aguilar J, Koche R, Lowe SW, Pe'er D, Shia J, Massagué J (2020). L1CAM defines the regenerative origin of metastasis-initiating cells in colorectal cancer. Nature Cancer.

[95] Rui F, Abdalkareem EA, Huat LB, Yin KB (2022). Clusterin mRNA silencing reduces cell proliferation, inhibits cell migration, and increases CCL5 expression in SW480, SW620, and Caco2 cells. Turkish Journal of Biochemistry.

[96] Tape CJ (2023). Plastic persisters: Revival stem cells in colorectal cancer. Trends in Cancer.

[97] Nusse YM, Savage AK, Marangoni P, Rosendahl-Huber AKM, Landman TA, de Sauvage FJ, Locksley RM, Klein OD (2018). Parasitic helminths induce fetal-like reversion in the intestinal stem cell niche. Nature.

[98] Qin X, Cardoso Rodriguez F, Sufi J, Vlckova P, Claus J, Tape CJ (2023). An oncogenic phenoscape of colonic stem cell polarization. Cell.

[99] De Palma FDE, D'Argenio V, Pol J, Kroemer G, Maiuri MC, Salvatore F (2019). The molecular hallmarks of the serrated pathway in colorectal cancer. Cancers.

[100] Guinney J, Dienstmann R, Wang X, de Reyniès A, Schlicker A, Soneson C, Marisa L, Roepman P, Nyamundanda G, Angelino P, Bot BM, Morris JS, Simon IM, Gerster S, Fessler E, De Sousa E, Melo F, Missiaglia E, Ramay H, Barras D, Homicsko K, Maru D, Manyam GC, Broom B, Boige V, Perez-Villamil B, Laderas T, Salazar R, Gray JW, Hanahan D, Tabernero J, Bernards R, Friend SH, Laurent-Puig P, Medema JP, Sadanandam A, Wessels L, Delorenzi M, Kopetz S, Vermeulen L, Tejpar S (2015). The consensus molecular subtypes of colorectal cancer. Nature Medicine.

[101] Solé L, Lobo-Jarne T, Álvarez-Villanueva D, Alonso-Marañón J, Guillén Y, Guix M, Sangrador I, Rozalén C, Vert A, Barbachano A, Lop J, Salido M, Bellosillo B, García-Romero R, Garrido M, González J, Martínez-Iniesta M, López-Arribillaga E, Salazar R, Montagut C, Torres F, Iglesias M, Celià-Terrassa T, Muñoz A, Villanueva A, Bigas A, Espinosa L (2022). p53 wild-type colorectal cancer cells that express a fetal gene signature are associated with metastasis and poor prognosis. Nature Communications.

[102] Regan JL, Schumacher D, Staudte S, Steffen A, Lesche R, Toedling J, Jourdan T, Haybaeck J, Mumberg D, Henderson D, Győrffy B, Regenbrecht CRA, Keilholz U, Schäfer R, Lange M (2021). RNA sequencing of long-term label-retaining colon cancer stem cells identifies novel regulators of quiescence. iScience.

[103] Moorman, A., Cambuli, F., Benitez, E., Jiang, Q., Xie, Y., Mahmoud, A., Lumish, M., Hartner, S., Balkaran, S., Bermeo, J., Asawa, S., Firat, C., Saxena, A., Luthra, A., Sgambati, V., Luckett, K., Wu, F., Li, Y., Yi, Z., Masilionis, I., Soares, K., Pappou, E., Yaeger, R., Kingham, P., Jarnagin, W., Paty, P., Weiser, M., Mazutis, L., D’Angelica, M., Shia, J., Garcia-Aguilar, J., Nawy, T., Hollmann, T., Chaligné, R., Sanchez-Vega, F., Sharma, R., Pe’er, D., & Ganesh, K. (2023). Progressive plasticity during colorectal cancer metastasis. *bioRxiv*, 2023.2008.2018.553925. 10.1101/2023.08.18.553925

[104] Zheng W, Yao M, Wu M, Yang J, Yao D, Wang L (2020). Secretory clusterin promotes hepatocellular carcinoma progression by facilitating cancer stem cell properties via AKT/GSK-3β/β-catenin axis. Journal of Translational Medicine.

[105] Arumugam P, Samson A, Ki J, Song JM (2017). Knockdown of clusterin alters mitochondrial dynamics, facilitates necrosis in camptothecin-induced cancer stem cells. Cell Biology and Toxicology.

[106] Álvarez-Varela A, Novellasdemunt L, Barriga FM, Hernando-Momblona X, Cañellas-Socias A, Cano-Crespo S, Sevillano M, Cortina C, Stork D, Morral C, Turon G, Slebe F, Jiménez-Gracia L, Caratù G, Jung P, Stassi G, Heyn H, Tauriello DVF, Mateo L, Tejpar S, Sancho E, Stephan-Otto Attolini C, Batlle E (2022). Mex3a marks drug-tolerant persister colorectal cancer cells that mediate relapse after chemotherapy. Nature Cancer.

[107] Fodde R, Verhagen M, Joosten R, Schmitt M, Sacchetti A, Choi J, Välimäki N, Aaltonen L, Augenlicht L (2023). Paneth cells as the origin of intestinal cancer in the context of inflammation. Research Square.

[108] Chen L, Dupre A, Qiu X, Pellon-Cardenas O, Walton KD, Wang J, Perekatt AO, Hu W, Spence JR, Verzi MP (2023). TGFB1 induces fetal reprogramming and enhances intestinal regeneration. bioRxiv.

[109] Sharif, I., Simon, A., Ernesta, N., Nalle, P., Ashish, K., Daniel, B., Anna, W., Tuure, S., Anne, J., Alessandro, O., Markku, V., Kirsi, H. P., Kim, B. J., Menno, O., & Pekka, K. (2021). Fetal-like reversion in the regenerating intestine is regulated by mesenchymal Asporin. *bioRxiv*, 2021.2006.2024.449590. 10.1101/2021.06.24.449590

